# Death by a thousand cuts: the challenges and diverse landscape of lignocellulosic hydrolysate inhibitors

**DOI:** 10.3389/fmicb.2014.00090

**Published:** 2014-03-14

**Authors:** Jeff S. Piotrowski, Yaoping Zhang, Donna M. Bates, David H. Keating, Trey K. Sato, Irene M. Ong, Robert Landick

**Affiliations:** DOE Great Lakes Bioenergy Research Center, University of Wisconsin-MadisonMadison, WI, USA

**Keywords:** cellulosic biofuels, lignocellulosic hydrolysate inhibitors, systems biology, chemical genomics, metabolic modeling, ethanologens

## Abstract

Lignocellulosic hydrolysate (LCH) inhibitors are a large class of bioactive molecules that arise from pretreatment, hydrolysis, and fermentation of plant biomass. These diverse compounds reduce lignocellulosic biofuel yields by inhibiting cellular processes and diverting energy into cellular responses. LCH inhibitors present one of the most significant challenges to efficient biofuel production by microbes. Development of new strains that lessen the effects of LCH inhibitors is an economically favorable strategy relative to expensive detoxification methods that also can reduce sugar content in deconstructed biomass. Systems biology analyses and metabolic modeling combined with directed evolution and synthetic biology are successful strategies for biocatalyst development, and methods that leverage state-of-the-art tools are needed to overcome inhibitors more completely. This perspective considers the energetic costs of LCH inhibitors and technologies that can be used to overcome their drain on conversion efficiency. We suggest academic and commercial research groups could benefit by sharing data on LCH inhibitors and implementing “translational biofuel research.”

## Introduction

Lignocellulosic biofuels offer the promise of sustainable, domestically produced fuels with favorable carbon balances. Fast-growing grasses like *Miscanthus* and agricultural residues provide fermentable sugars at lower energy and fertilizer costs than grains (Schmer et al., 2008), making them preferable feedstocks for advanced biofuels. Cellulosic ethanol is an obvious next-generation biofuel to implement given its production and delivery infrastructures are compatible with existing fuels.

Central to the success of cellulosic ethanol is efficient conversion of biomass-derived sugars to ethanol by microbes such as *Saccharomyces cerevisiae*, *Escherichia coli*, and *Zymomonas mobilis* (Alper and Stephanopoulos, 2009; Lau et al., 2010; Yang et al., 2010a). Under optimal conditions, these microbes are powerful ethanologens; however, lignocellulosic hydrolysates (LCH) and industrial scale fermentation tanks are not optimal conditions. Thermal, osmotic, and ethanol stresses are just some of the environmental factors that inhibit fermentation and reduce yield (Attfield, 1997; Gibson et al., 2007; Jin et al., 2013). Industrial microbes are pushed to the limits of stress tolerance to make biofuel production energetically favorable.

Although environmental stressors limit yields in present day ethanol facilities, cellulosic biomass conversion comes with new challenges. Specifically, LCH inhibitors, a group of small, bioactive molecules can significantly reduce conversion efficiency. LCH inhibitors such as aliphatic acids, furans, and phenolics are released or condensed from cellulose and hemicellulose during pretreatment and hydrolysis (Larsson et al., 1999, 2000; Yang et al., 2010a); however, chemical residues from newer hydrolysis strategies and synergies with biofuel end products (ethanol, isobutanol) are less well studied. Removal of these inhibitors can be expensive and may reduce titers of fermentable sugars; some estimates suggest that detoxification can remove up to 26% of total fermentable sugars (Larsson et al., 1999). Thus, a preferred strategy is to develop microbial strains with properties that minimize the effects of LCH inhibitors on biofuel yields.

With commercially available industrial stains that are robust to thermal and ethanol stress (e.g., Ethanol Red, Fermentis, Milwaukee, WI, USA), recent attention has been directed to overcoming the challenge of LCH inhibitors. These compounds are ubiquitous in hydrolysates, and their abundance and composition depends on pretreatment (Chundawat et al., 2010), feedstock (Klinke et al., 2004; Almeida et al., 2007), and seasonality (Bunnell et al., 2013; Greenhalf et al., 2013). Given their chemical diversity, these compounds can target many cellular processes. LCH inhibitors can also generate a substantial cellular energy drain. Cells have evolved to detoxify, excrete inhibitors, or repair the resultant cellular damage fast enough to reproduce. However, evolved coping mechanisms may also negatively affect the efficiency of conversion by competing for cellular resources (Bellissimi et al., 2009; Miller et al., 2009). Although it is in the microbe's best interest to use its resources to limit the effects of LCH inhibitors and maintain cellular viability, this may be reducing biofuel production. In this perspective, we consider the diversity and cellular costs of LCH inhibitors from traditional and novel pretreatment and hydrolysis strategies, describe new technologies and their application to strain development, and finally identify key needs of the cellulosic biofuel community that will empower “translational biofuel research” to take discoveries quickly to industrial scale.

## Diversity of fermentation inhibitors

Prior to microbial conversion of lignocellulosic sugars into biofuel, biomass must be deconstructed into monomeric sugars by enzymatic or chemical hydrolysis. This hydrolysis step is often preceded by a pretreatment step that expands the plant fibers and allows cellulolytic enzymes access to the polysaccharide matrices. The resulting hydrolysates are complex, ill-defined mixtures that include sugars and a diversity of bioactive molecules (Table 1). Small acids and phenolic compounds are released from cellulose and hemicelluloses during hydrolysis and furans arise from the dehydration of pentose and hexose monomers (Klinke et al., 2004). Pretreatments such as acid hydrolysis, steam explosion or NH_3_ expansion each impart their own “profile” of LCH inhibitors. For example, AFEX (Ammonia Fiber EXpansion) uses high-pressure/temperature ammonia to alter the cellulose matrix to allow hydrolysis by cellulases (Lau and Dale, 2009), and this produces amide versions of inhibitors (e.g., feruloyl amide from ferulic acid) with potentially new biological properties (Chundawat et al., 2010).

**Table 1:** **Classes of lignocellulosic hydrolysate (LCH) inhibitors and their described modes of toxicity**.

**Inhibitors**	**Mode of action**	**References**
**LIGNOCELLULOSE DERIVED**		
**Small acids**		
Acetic acid, formic acid, levulic acid	Decreases cellular pH,	Sinha, 1986; Cherrington et al., 1990; Holyoak et al., 1996; Stratford and Anslow, 1998; Bellissimi et al., 2009; Ullah et al., 2012; Ding et al., 2013
	Decreases cellular ATP,	
	Inhibits macromolecule biosynthesis,	
	Inhibits DNA synthesis/repair,	
	Inhibits glycolytic enzymes	
**Furans**		
Furfural, HMF, 2-furoic acid	Damages membranes,	Ingram, 1976; Hadi and ShahabuddinRehman, 1989; Khan et al., 1995; Zaldivar and Ingram, 1999; Modig et al., 2002; Miller et al., 2009; Allen et al., 2010; Wang et al., 2013
	Oxidative damage,	
	Damages nucleic acids,	
	Damages proteins,	
	Limits sulfur assimilation,	
	Reduces NADH/NADPH pools,	
	Inhibits enzymes	
**Phenolics**		
Ferulic acid, coumaric acid	Damages membranes,	Krebs et al., 1983; Mikulášová et al., 1990; Verduyn et al., 1992; Chambel et al., 1999; Fitzgerald et al., 2004; Iwaki et al., 2013; Nguyen et al., 2014
Vanillin, Syringealdehyde	Decreases cellular pH,	
Coniferyl alcohol, Eugenol	Decrease cellular ATP,	
Acetovanillin, Feruloyl amide, Coumaryl amide	Inhibits translation,	
	Oxidative damage,	
	Denatures proteins,	
	Damages cytoskeleton,	
	DNA mutagenesis,	
	Induces apoptosis	
**PROCESS DERIVED INHIBITORS**		
**Ionic liquids**		
1-Ethyl-3-methylimidazolium-Ac	Unknown	Docherty and Kulpa, 2005
**Surfactants**		
Triton-X, Tween	Damages membranes	King et al., 1991; Laouar et al., 1996
**Metal ions**		
Copper, Sodium, Zirconium	Damages membranes, nucleic acids, and enzymes	Shapiro and Ling, 1998; Schmitt and Tampé, 2002
γ-valerolactone	Unknown	Luterbacher et al., 2014
**End product inhibitors**		
Ethanol	Damages membranes	Nagodawithana and Steinkraus, 1976; Dombek and Ingram, 1984; Alexandre et al., 1994; Ibeas and Jimenez, 1997; Huffer et al., 2011
Isobutanol	Damages DNA	
	Inhibits enzymes	

Besides these common inhibitors, residual pretreatment chemicals may complicate fermentation. Ionic liquids are pretreatment and hydrolysis solvents, but are toxic to many microorganisms (Docherty and Kulpa, 2005; Ouellet et al., 2011). Alkaline hydrogen peroxide (AHP) pretreatment limits the production of furans; however this method introduces significant amounts of Na^+^ from NaOH, which can cause osmotic stress (Sato et al., 2014). Copper(II) 2,2′-bipyridine is a catalyst that enhances AHP pretreatment by reducing the H_2_O_2_ requirement (Li et al., 2013), but copper is toxic to most microbes. Next-generation pre-treatments and hydrolysis methods like γ-valerolactone (Luterbacher et al., 2014), surfactants (Sindhu et al., 2013), zirconium phosphate catalysts (Gliozzi et al., 2014), and other incipient hydrolysis technologies may imbue hydrolysates with novel toxicities and synergisms with common inhibitors.

Biofuel end-products themselves are inhibitory. Ethanol can directly damage cellular membranes, DNA, as well as inhibit enzymes (Nagodawithana and Steinkraus, 1976; Dombek and Ingram, 1984; Alexandre et al., 1994; Ibeas and Jimenez, 1997; Huffer et al., 2011). The ethanologens *S. cerevisiae* and *Z. mobilis* are not immune to ethanol toxicity at high concentrations (Carmona-Gutierrez et al., 2012; Yang et al., 2013). Advanced biofuels like isobutanol are toxic at significantly lower concentrations than ethanol (Brynildsen and Liao, 2009; Atsumi et al., 2010; Huffer et al., 2011; Minty et al., 2011). Inhibition by end products has been an area of research interest (Baez et al., 2011; McEwen and Atsumi, 2012; Zingaro et al., 2013), and ethanol tolerance is a pre-requisite for all industrial yeast.

Effects of inhibitors at a minimum can be additive, but an even greater concern is the potential for synergy between LCH inhibitors and fermentation condition, including high osmolarity and absence of O_2_. Some studies have described synergies between acetic acid, furfural, and phenolics in yeast (Oliva et al., 2006; Ding et al., 2011), but a comprehensive evaluation of synergisms between compounds and conditions on both growth rate and fermentation will be essential. Such assessment will be a massive undertaking that will also require defined synthetic hydrolysate media to permit meaningful definition of minimum inhibitory concentrations (MICs) of the individual inhibitors, and how this value will change in combination with other LCH inhibitors (synergy/antagonism) and fermentation conditions. Nevertheless, documenting interactions between inhibitors on sugar conversion is crucial to prioritizing future research for improved biofuel microbes.

## Small molecule inhibitors deplete cellular resources

**Figure 1:**
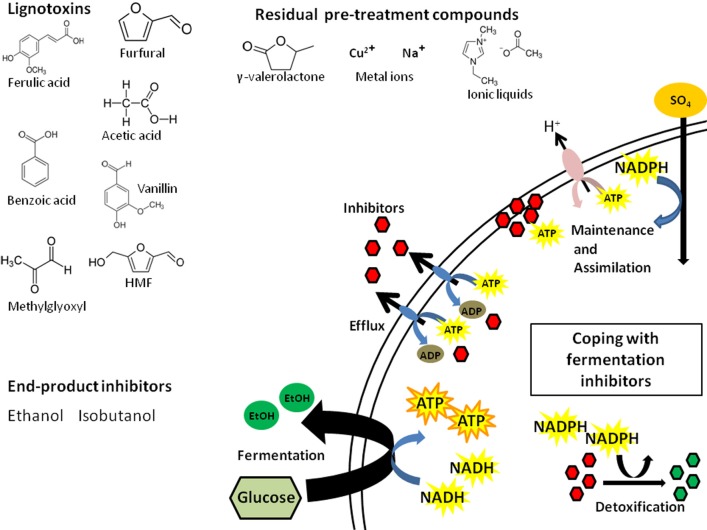
**Inhibitor classes and the cellular energy consequences of LCH inhibitors**. Presented are examples from three main classes of inhibitors and the ways cells can cope with these: efflux via pumps, detoxification via enzymes, and repair of the damage caused by the compounds. Each coping strategy comes at the expense of cellular energy that is diverted from the stores used to maintain cell integrity by chemiosmotic exchange and assimilation as well as energy required for fermentation of sugars to fuel.

LCH inhibitors directly affect biofuel yield as well as the production rate, which can extend fermentation time and result in higher operating costs. In simplest terms, these inhibitors affect conversion efficiency by depleting cellular energy resources (e.g., ATP, NADH, NADPH; Figure 1). Some inhibitors can act broadly and damage key enzymes of fermentation pathways (Modig et al., 2002). The coping mechanisms available to the biofuel microbes fall into 4 main categories: (i) detoxification, (ii) efflux, (iii) repair, or (iv) tolerance. The first three are of most concern given their effects on cellular energy and resources.

Detoxification and efflux are the most well characterized mechanisms of inhibitor tolerance in microbes, with deep literature not only from biofuel research but also in the medical literature from a wealth of antibiotic and pharmaceutical research. Detoxification is the major route of tolerance for aldehydes in both bacteria and yeast. Reduction of compounds like furfural and vanillin to less toxic alcohols by NADH/NADPH dependent reductases occurs in ethanol fermentation using *S. cerevisiae* and *E. coli* (Jarboe, 2011). Oxidoreductase expression significantly increases in yeast and *E. coli* in the presence of aldehydes (Liu et al., 2008; Wang et al., 2011), depleting cellular NADH/NADPH. This results in inhibition of NADPH-dependent processes (e.g., assimilation of sulfur) leading ultimately to slower conversion of sugars (Miller et al., 2009). Interestingly, yields can be increased by disabling the detoxification pathway, suggesting that tolerance may be more energetically efficient than detoxification (Wang et al., 2013). Alternatively, changing the source of reducing equivalent for aldehyde detox from NADPH to NADH also can improve biofuel yield (Wang et al., 2013).

Efflux is mediated by ATP-dependent trans-membrane pumps that selectively or non-selectively pump out toxic compounds usually at the cost of 1 ATP per molecule (Shapiro and Ling, 1998; Schmitt and Tampé, 2002). The yeast *S. cerevisiae* has 29 different ATP-binding cassette (ABC) efflux transporters (Decottignies and Goffeau, 1997) and 5% of the *E. coli* genome is composed of genes with ABC-transporter domains, many involved in efflux (Linton and Higgins, 1998). In both yeast and *E. coli*, expression of transporters increases in response to LCH inhibitors (Schüller et al., 2004; Lee et al., 2012; Schwalbach et al., 2012). The yeast weak acid response is mediated by increased expression of ABC-transporters (e.g., Pdr1p and Pdr5p) (Schüller et al., 2004; Pereira Rangel et al., 2010). As long as cells are exposed to LCH inhibitors, a significant fraction of cellular ATP will be diverted to efflux pumps. Of particular importance for overall efficiency of LCH conversion, ATP-depletion may have a disproportionate effect on xylose conversion compared to glucose conversion because xylose produces less cellular energy per molecule transported (Matsushika et al., 2013). Bellissimi et al. (2009) found that acetic acid could specifically inhibit xylose fermentation, but that this effect could be reversed with glucose addition. The authors posit that ATP generated from xylose fermentation cannot match ATP depletion from the weak acid response and efflux/proton pumps used to maintain cellular pH. Inhibitors that require ATP-dependent coping mechanisms can directly reduce xylose conversion. Some inhibitors can be particularly draining, affecting both NADPH and ATP pools. Ask et al. (2013) found that furfural and HMF not only reduced cellular NAPDH in *S. cerevisiae*, but also elicited increased expression of ATP-dependent efflux pumps Pdr5p and Yor1p. This suggests that coping with furans requires both NADPH dependent detoxification and ATP-dependent efflux.

An unanswered question is whether the ATP-dependent action of efflux pumps has a net positive (by inhibitor removal) or net negative (by energy consumption) effect on biofuel production. Although the answer may vary by inhibitor, because some inhibitors like aldehydes are more damaging to cells than others, a general test of the positive or negative consequences of efflux pumps for biofuel yield will help advance strategies for biofuel microbe design.

The cellular energy costs of maintenance and repair are more difficult to quantify but could account for significant energy loss. If cells can repair the damage caused by fermentation inhibitors quickly, then fermentation may proceed. Inhibitors can acidify cells (Verduyn et al., 1992), damage cellular membranes (Russell, 1992), DNA (Allen et al., 2010), and individual proteins (Modig et al., 2002). Repairing structures requires biogenesis; this comes at the expense of ATP, NADPH, carbon, and nitrogen. Maintaining pH is mediated by ATP-dependent proton pump Pma1p; and ATP cost under acidic conditions is the primary cause of reduced cellular growth (Verduyn et al., 1992; Ullah et al., 2012). Biogenesis requires NADPH-dependent assimilation of nutrients like sulfur, which is drained by repair enzymes. Phenolics and furans can damage membranes, requiring more energy to maintain the proton gradient required for basic metabolism (Ding et al., 2012; Schwalbach et al., 2012; Stratford et al., 2013). Growth and sugar conversion will be slowed as resources are diverted to maintenance and repair. Given that hydrolysates contain a mixture of inhibitors with diverse modes of action requiring all of these coping mechanisms simultaneously, the energy drain from fermentation inhibitors is truly death by a thousand cuts.

## Adapting to the changing landscape of LCH inhibitors

Strain development is an economical route to deal with LCH inhibitors. Resistance to the suite of inhibitors requires complex response of many cellular systems, and as such is not easily conferred by engineering of individual genes. Moreover, inhibitor pools can vary between hydrolysate preparations, thus even the most robust strains in ammonia-pretreated hydrolysate may wither in dilute acid pretreated hydrolysates. It is unlikely that one strain will be optimal for all conditions. The reality is that microbial strains will need to be tailored to specific hydrolysates through engineering and directed evolution. Accelerating this process is crucial to making new cellulosic technologies industrially viable.

The tools of systems biology can give a detailed view of the microbial stress response (Jozefczuk et al., 2010; Lee et al., 2011). Transcriptomic, proteomic, and metabolomic responses to inhibitors can be tracked in detail, and this “multiomic” approach can give high-resolution insight to the global cellular consequences (Miller et al., 2009; Yang et al., 2010b; Schwalbach et al., 2012; Skerker et al., 2013; Yang et al., 2013). Advanced techniques such as ribosome profiling can give a view into relationships between transcription and protein abundance in the presence of LCH inhibitors (Ingolia et al., 2009; Brar et al., 2012). Metabolic and flux-balance models are valuable in determining energy balances within cells (Varma and Palsson, 1994; Fiaux et al., 2003; Jin and Jeffries, 2004; Herrgård et al., 2008; Taymaz-Nikerel et al., 2010). These models combined with a systems biology view of protein and gene expression can be used to identify key energetic bottlenecks as targets for engineering. Recently, Wei et al. (2013) demonstrated an elegant way to overcome the redox cofactor imbalance in yeast designed to ferment xylose by engineering acetate metabolism from *E. coli* into *S. cerevisiae*. The authors combined an acetate utilization pathway that consumes NADH with a xylose utilization pathway that produces NADH to overcome the redox imbalance of engineered xylose fermentation. The resultant strain has both better xylose conversion and the ability to detoxify acetate (Wei et al., 2013). Detailed accounting of ATP and NAD(P)H in the presence of LCH inhibitors and industrial conditions will be necessary to disentangle and understand points for rational engineering of microbial catalysts.

Model biofuel microbes like *S. cerevisiae* and *E. coli* benefit from well-developed suites of functional genomics resources, such as deletion mutant or overexpression collections (Giaever et al., 2002; Baba et al., 2006; Kitagawa et al., 2006). These tools have revealed effects of some inhibitors such as furfural (Gorsich et al., 2006), vanillin (Endo et al., 2008; Iwaki et al., 2013), and acetic acid (Mira et al., 2010). Genome-wide mutant collections also allow powerful studies of inhibitors via “chemical genomics” (Giaever et al., 2004; Parsons et al., 2006; Ho et al., 2011). This new tool in the multiomic arsenal, when combined with the information in genetic interaction networks (Butland et al., 2008; Costanzo et al., 2010), can allow precise predictions of the cellular targets of fermentation inhibitors. Recently, Skerker et al. (2013) used a chemical genomics approach to discover a previously undescribed inhibitor in acid pretreated hydrolysate, methyl glyoxal (MG), and identified mutations that confer MG resistance. Chemical genomics can be used to identify the chemical biological signatures within hydrolysates and mutations conferring resistance, but more broadly can serve as a “biological fingerprinting” technique for hydrolysate to identify variation in production, and as a method to benchmark the biological properties of novel hydrolysates. Resources such as the MoBY-ORF collections (Ho et al., 2009; Magtanong et al., 2011), which are barcoded plasmids collections carrying nearly all *S. cerevisiae* used to assess the effects of increased gene dose and gene complementation, could be used with industrial, wild, and engineered yeast to identify genetic interactions within diverse yeast strains. Combined with traditional selection for resistance and directed evolution, systems biology tools offer great potential for strain development.

Further, new tools are now available that can accelerate strain development. Genome editing and optimization techniques such as CRISPR/Cas9 and MAGE allows rapid, detailed genome editing in both bacteria and eukaryotes (Wang et al., 2012; Cong et al., 2013; DiCarlo et al., 2013; Gilbert et al., 2013). Next steps in strain development will be tuning genomes to inhibitor tolerance by parallel disruption or activation of inhibitor responsive genes using CRISPR/Cas9-based systems. Additionally, large-scale gene synthesis can be used to identify genes that not only aid xylose utilization, but also confer inhibitor tolerance. Systems biology tools for biocatalyst development are coalescing to a pipeline that can keep pace with the changing landscape of fermentation inhibitors, allowing for the rapid tailoring of ethanologens with robust and efficient sugar to biofuel conversion from any new hydrolysate.

## Necessary tools to meet a common goal

Much like the interface between commercial and academic drug discovery communities, applying next-generation biofuel technologies will require cooperative translational research. Academic biofuel communities are developing advanced system biology techniques whereas the commercial community excels at scale-up commercialization. However, similar to drug discovery, these two groups are often isolated in their research. In both cases, public-private partnerships such as the NIH translational medicine initiatives (Zerhouni, 2003) and NCERC industrial partnerships (http://www.siue.edu/ethanolresearch/), as well as shared computational resources like Kbase (http://www.kbase.us/) can help bridge the divide.

How can researchers and funding agencies best enhance collaboration? A key advance would be greater data sharing about LCH inhibitors. Diverse hydrolysates and their respective inhibitors are major variables in the field of cellulosic biofuel production, but detailed information on specific hydrolysate compositions is not broadly available. Comprehensive efforts to identify all major LCH components and inhibitors across feedstocks and hydrolysis treatments are needed and will require a central repository of LCH data that includes data on feedstocks, pretreatment, hydrolysis, nutrients, and inhibitor. Chemical genomic profiling could be used to generate “biological fingerprints” of hydrolysates to allow comparisons among hydrolysates and identify the biological effects of LCH components by standard analytical methods. A central resource would allow researchers to compare the composition and biological fingerprints of new hydrolysates with existing knowledge about tolerant microbes for further strain development. The DOE's Systems Biology Portal, KBase, offers the best outlet for community resources, and could serve as the authority of the response of biocatalysts to LCH inhibitors with open-source, community-developed analytical tools for chemical genomics datasets.

## Conclusions

LCH inhibitors are major barriers for cellulosic biofuels. The cellular energy costs of coping with these compounds are a significant drain on the already thin margins of biofuel production. However, the increasingly powerful tools of systems biology can be used to gain a detailed understanding of the cellular consequences of individual and mixtures of fermentation inhibitors, which will serve as a basis for rational engineering of customizable microbes. The biofuel research community would benefit from shared computational and database resources that can improve communication between the academic and commercial sides of biofuels.

### Conflict of interest statement

The authors declare that the research was conducted in the absence of any commercial or financial relationships that could be construed as a potential conflict of interest.
